# Melanoma of the Right Foot Simulating Kaposi's Disease

**DOI:** 10.1155/2015/750491

**Published:** 2015-10-08

**Authors:** K. A. Kouassi, K. Kassi, K. Kouamé, M. A. Oussou, I. Kouassi, I. P. Gbery, E. J. Ecra, A. Sangare, C. Ahogo, M. Kaloga, P. Yoboue, J. M. Kanga

**Affiliations:** ^1^Department of Dermatology and Infectiology, University of Félix Houphouët Boigny of Abidjan, BP V 166, Abidjan, Côte d'Ivoire; ^2^Department of Dermatology, Alassane Ouattara University, Bouake, Côte d'Ivoire

## Abstract

Melanoma is a malignant tumor rarely being described in sub-Saharan Africa. We reported an unusual and atypical clinical presentation. It was a 59-year-old patient who was hospitalized for a monomelic black tumor evolving for 10 years. Histopathological examination confirmed the melanocytic origin of this tumor. Paraclinical assessment did not find any visceral metastasis. A partial resection of the tumor was performed. The patient left the hospital against medical consent due to lack of technical facilities. The delay in the consultation and the lack of knowledge of melanoma by doctors and patients might contribute to the severity and the difficulties of its management.

## 1. Background

Melanoma is a malignant skin tumor developed from melanocyte cells. Although melanoma represents only 5% of all occurrences of skin cancers in the USA, it is responsible for 75% of all skin cancers-related deaths [1]. It is mostly linked to lymph node metastasis associated with unusual metastasis location in some organs [2, 3].

This black skin tumor which was rare in the past has become more and more observed in black Africans associated with unusual metastasis locations [4, 5]. Here, we reported a case of melanoma associated with atypical and unusual clinical presentation.

## 2. Case Report

**Figure 1:**
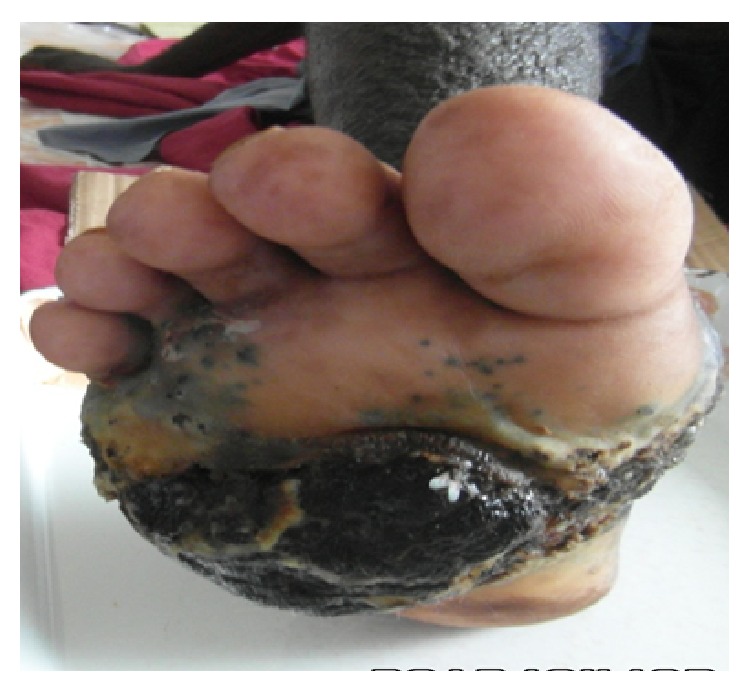
Nodular melanoma of the sole of the right foot.

**Figure 2:**
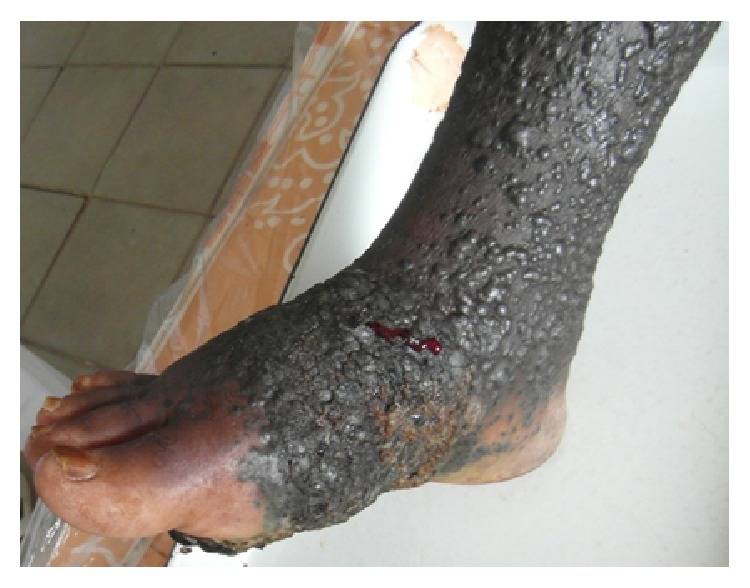
Cutaneous in-transit metastasis of a nodular melanoma of the right foot.

**Figure 3:**
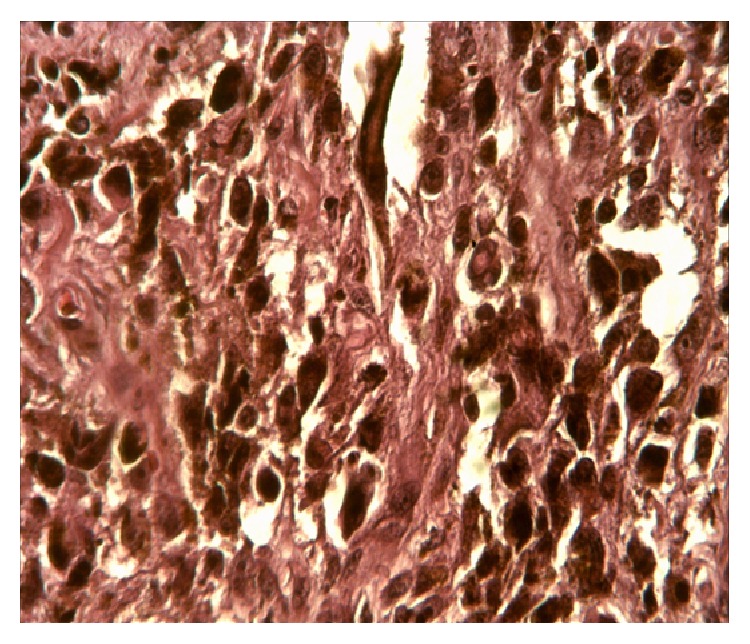
Histopathological aspect of pigmented melanoma, stage V of Clark and Mihm classification.

It was a 59-year-old patient who was hospitalized for black skin tumor. This tumor started by a black macula located on the sole of the right foot associated with pruritus. One week later, the macula became ulcerated with flowing of a purulent liquid. Days later, it budded and extended progressively and was associated with black papules on all the skin surface of the right foot. In addition, the patient revealed that he is a tobacco user (60 boxes of cigarettes per year). The clinical examination showed growing and ulcerated tumor which measured 30 centimeters in diameter.It was a bleeding and painful tumor which invaded laterally the heel and in depth the interosseous muscles (Figure 1). This black skin tumor presented hyperpigmented nodule and papules (called permeating nodules) on its surface without healthy skin intervals (Figure 2), associated with large inguinal lymphadenopathy. In addition, blackish urines were observed from the patient during hospitalization, suspecting a presence of melanin. Therefore melanoma was evoked. Histopathological examination of tumor biopsy confirmed the diagnosis of pigmented melanoma stage V according to Clark and Mihm classification (Figure 3). The rest of the paraclinical examination without MRI was normal including HIV testing after informed consent. A palliative surgical treatment was decided in our case. But our patient did not accept the treatment option and he left the hospital without medical consent.

### 2.1. Comments

Unusual melanoma metastases were already reported in the literature. In fact, previous work conducted by Ben Hadj Hamida and by EL Otmami has reported some atypical melanoma metastasis. There were orbitary and bone melanoma metastases, respectively [2, 3].

These different studies revealed the severity and the ubiquitous character of this malignant skin cancer. The acral location of melanoma represents the most frequent form of melanoma in black Africa, although the etiologic factors remained unclear. The nodular form of melanoma ulcerated or not has bad prognosis due to rapid skin infiltration and occurrence of lymph nodes metastasis. This metastasis occurs by blood vessels or by local way [4, 5]. In our case, this classical presentation was observed with the presence of permeating nodule and lymph nodes metastasis on the right lower limb associated with a hard infiltrated edema. The clinical expression looked like Kaposi's disease. Meanwhile, because of the pasty and blackish substance associated with blackish urines observed during hospitalization, we thought about melanoma. Furthermore, the onset of melanoma depends on intrinsic factors such as sex (men are more concerned than women) and age (over 50 years) and heredity factors [6]. Our patient was a 59-year-old male, but no similar cases were found in his family. The cases of monomelic melanoma caused by UV rays published in 2004 by Zouhair et al. [7] and treated by local chemotherapy were similar to our case report. Meanwhile, the presence of lymph nodes metastasis and the secondary localization in the patient's urinary tracts led to treatment difficulty. In our patient, the melanoma location on the skin and in the lymph nodes without clinical organ metastasis could explain the relative conservation of the general body aspect and the longer disease course (15 years). But the narrowness of our medical technical platform such as the lack of MRI (to explore visceral metastasis), X-ray therapy, and target therapy access led to bad prognosis.

## 3. Conclusion 

Melanoma remains the most dangerous skin cancer in its frequent locations as well as in the unusual locations. This reported clinical case pointed out the difficulty in both diagnosis and treatment of the sole melanoma in sub-Saharan Africa. Therefore it seems important to set up preventive measures for these cancers including early treatment strategies.
